# Ag(e)ing and Degradation of Supercapacitors: Causes, Mechanisms, Models and Countermeasures

**DOI:** 10.3390/molecules28135028

**Published:** 2023-06-27

**Authors:** Xuecheng Chen, Yuping Wu, Rudolf Holze

**Affiliations:** 1 Faculty of Chemical Technology and Engineering, West Pomeranian University of Technology, Piastów Ave. 42, 71-065 Szczecin, Poland; xchen@zut.edu.pl; 2 School of Energy and Environment, Southeast University, Nanjing 210096, China; wuyp@fudan.edu.cn; 3 Chemnitz University of Technology, D-09107 Chemnitz, Germany; 4 Institute of Chemistry, Saint Petersburg State University, St. Petersburg 199034, Russia; 5 State Key Laboratory of Materials-Oriented Chemical Engineering, School of Energy Science and Engineering, Nanjing Tech University, Nanjing 211816, China

**Keywords:** supercapacitor, ultracapacitor, electrochemical capacitor, electrochemical double layer capacitor, aging, ageing, degradation, pseudocapacitive

## Abstract

The most prominent and highly visible advantage attributed to supercapacitors of any type and application, beyond their most notable feature of high current capability, is their high stability in terms of lifetime, number of possible charge/discharge cycles or other stability-related properties. Unfortunately, actual devices show more or less pronounced deterioration of performance parameters during time and use. Causes for this in the material and component levels, as well as on the device level, have only been addressed and discussed infrequently in published reports. The present review attempts a complete coverage on these levels; it adds in modelling approaches and provides suggestions for slowing down ag(e)ing and degradation.

## 1. Introduction

Among the various properties and parameters describing the performance of a supercapacitor, the stored energy, the power (current) possibly delivered and received and the internal resistance ESR (electrical series resistance) are most relevant for the use and the proper operation of such a device. In device-related terms, the capacitance and the ESR are commonly considered when evaluating a device. Although there does not appear to be a standard, it seems that a capacitance loss of 20% compared with the initial value and an increase of the ESR by 100% are the failure criteria that describe a device as worn out. The statement in [1] suggesting that the 80% criterion was set in the IEC 62391-1 standard attributed to [2] is misleading; the authors in the latter communication refer to this standard only because it describes the procedure of measuring the capacitance without proving any further criterion. Despite the claims praising the exceptional stability, i.e., constancy of said parameters, they tend to change over time and use, i.e., calendar ageing in terms of time passed and cyclic ageing in terms of run charge/discharge cycles can be noticed [3]. Both forms of ageing (the concept of spelling aging, also found in the literature, is not used in the following text) can be studied with different methods, but results may be different according to the various forms and different parameters used.

Presumably the most popular approach is running galvanostatic charge/discharge cycles (GCD) within a defined electrode potential window for an electrode (an electrode material) or a cell voltage window for a complete device. Comparison of the recovered charge (or, less frequently, energy, as discussed below in more detail) or the capacitance calculated from the recorded data for the first and the last cycle yields a percentage change, in most cases reported as capacity (the term capacitance is used as a synonym in many reports) retention. In earlier publications, this was termed degradation, with degradation reported in percentages appearing somewhat awkward. The term degradation is currently applied to material and device changes resulting in poorer performance. Recently, the effects of various factors on the degradation (not ageing) of supercapacitors have been analysed in a report that is somewhat difficult to understand [4]. At least for electrochemical double-layer capacitors (EDLC), ageing under cycling conditions was found to be faster than plain calendar ageing under similar conditions regarding applied voltage [5].

The same basic information on ageing can be obtained for both electrodes and devices from cyclic voltammograms. The inherent problems of this methodology have been previously discussed [6]. The obtained results can be considered as cyclic aging. Different from this, as already discussed elsewhere [3], calendar ageing can be examined simply by running GCDs or CVs with greater or lesser time intervals in between. Any degradation occurring in a material or in a device resulting in lowered performance, i.e., lower capacity, will be attributed to calendar aging. Once again, experimental conditions, particularly the state of charge and the environmental and device temperature, must be reported. In case the device is not just left at open cell voltage but kept connected to a monitoring circuit at a fixed voltage, the experiment can be classified as a “voltage hold” [7,8] or floating [9] test. According to research reports, signs of ageing may appear sooner in “voltage hold” tests than in cycling tests; for examples, see [7,8]. The “voltage hold” tests were deemed to be more demanding, i.e., performance losses were found earlier than with cycling procedures when exactly the same upper cell voltage limits were maintained in both approaches. The fact that in cycling tests the cells, particularly the positive electrodes, are exposed to voltage/potentials where accelerated degradation proceeds for shorter times than in the “voltage hold” experiments was proposed as an explanation. The current (in GCD) and the scan rate (in CV) applied in cycling tests escape this methodological comparison; the current applied during the few cycles between “voltage hold” periods hardly helps. While applying high cell voltages in “voltage hold” tests hardly poses an experimental challenge, high currents or scan rates applied in particular to devices with practically relevant capacities may overextend the practical capabilities of the employed instrumentation. In a re-examination of testing procedures for EDLC devices, the “voltage hold” test was proposed as a means to shorten evaluation times [10]. Carbon corrosion of the positive electrode, resulting in smaller surface area and higher oxygen content of the carbon electrode material, were observed. At the negative electrode, increases of surface area and pore volume were found. As a general conclusion, caution during comparison of ageing studies with different methods was recommended. In a slightly different approach, test conditions closely resembling practical use (defined after an extensive review of practical uses of EDLC supercapacitors) were applied [11]. The results were basically in agreement with the conclusions based on the above discussion of elevated temperatures significantly accelerating capacitance loss; the temperature effect was slightly more pronounced on the growth of the ESR. It must always be kept in mind that the actual temperature in a device depends on the environmental temperature, but can be significantly higher inside the device because of Joule heating inside caused by flowing current.

Electrochemical impedance measurements have been proposed as a further tool to monitor device degradation, i.e., ageing [12]. Degradation effects were reported in terms of changing impedance parameters. The claimed clear correlation of such changes with specific degradation phenomena is hard or impossible to find in the report. Application of the Mott–Schottky model has been recommended for selecting electrode potentials where impedance measurements should be made [13]. Calendar and cyclic ageing have been compared based on impedance measurements [14]. Based on the obtained results, it could be confirmed that both types of test affect supercapacitor performance differently. Impedance models (actually equivalent circuits) for EDLC-type supercapacitors have been critically compared, and a multi-pore model was recommended [15]. Using a simple Randles circuit [16], impedance measurement data from an EDLC device were fitted [17]. Unfortunately, no option to verify the quality of the fits was provided in the report; the list of fitted parameters does not even show units. A further approach to evaluating impedance data based on evolutionary programming has been applied for studies of degradation processes in EDLC supercapacitors [18]. Changes of microstructure and formation of deposits were noticed, with the ion transport inside the porous material being the process most negatively affected. Even early failure detection should be possible with this approach. In another study of activated carbon (AC) for EDLC application, structural changes negatively affected ion transport, particularly together with decreasing electronic conductivity and an electrochemically active surface were observed as the main causes of performance degradation [19]. Measurements of electric flicker noise have been indicated as a possible method of detecting changes in interfacial behaviour in supercapacitor cells, supplementing capacitance and ESR data [20]. Dilatometric measurements may help to gather information about volume changes of materials and components in devices [21]. A cell for the thermal analysis of supercapacitors has been described [22].

For comparison of data from different sources, further experimental conditions down to electrode thickness must be carefully taken into account; for a discussion, see [23], and in a more general context [24].

Further uses of the terms aging, ageing and degradation in reports on supercapacitors and their components can be observed. With some metal chalcogenides as active masses during the synthesis of the materials, ageing of the freshly synthesized material may be a part of the procedure; similar details have been mentioned with other, e.g., template-based synthetic procedures (see, e.g., [25,26,27,28,29,30,31]). This phenomenon is not considered here. Variations in the cation ratio of layered double hydroxides may also result in changes, referred to as degradation in [32]. Oxide layers on the surface of transition metal nitrides and oxynitrides studied as electrode materials show ageing behaviour during extended exposure to ambient air; this may in turn affect the capacitive performance of the material [33]. Thermal processes such as pyrolysis or carbonization are encountered when, e.g., polymers or biomass are converted into carbon materials. Why such a process is called degradation in, e.g., [34,35] remains unclear. The term degradation also appears in a report on chemical analysis during recycling of carbon materials for electrochemical energy storage and conversion devices [36]. To make matters even more complicated, unwanted changes in a carbon skeleton obtained initially from coconut shells at too-high temperatures were also referred to as degradation [37]. This concern also applies to changes of precursors in other synthetic procedures [38]. During synthetic procedures, templates may be used (see, e.g., [39,40,41]). When they are removed later in the synthetic procedure, this may be termed degradation (of the template). Again, these cases are not included here. With flexible and bendable electrodes and devices, degradation caused by bending is possible, e.g., cracks in the active layer or delamination from the current collector. Such phenomena are generally not addressed with the term ag(e)ing. Because reports on this type of degradation were part of the literature search output, they are mentioned only briefly in the following text. In a report on a conjugated supercapacitor, the addition of TiO_2_ resulted in “optimized retained energy upon aging” [42,43]. The meaning of aging could not be resolved; it may refer to the retention of stored energy upon storage of the device. When electrode degradation as a cause of device (i.e., a battery) ageing results in diminished capacitance, a current specified with respect to capacitance may actually grow when the absolute current is kept constant at a decaying capacitance. Thus, actual rates may grow during ageing experiments. As a correcting measure, current-corrected strategies have been proposed in battery research [44]. Similar considerations may apply in supercapacitor research. Whether imperfect electric contact between current collector and active mass should be referred to as cell degradation appears to be more a question of correct terminology [45].

**Figure 1:**
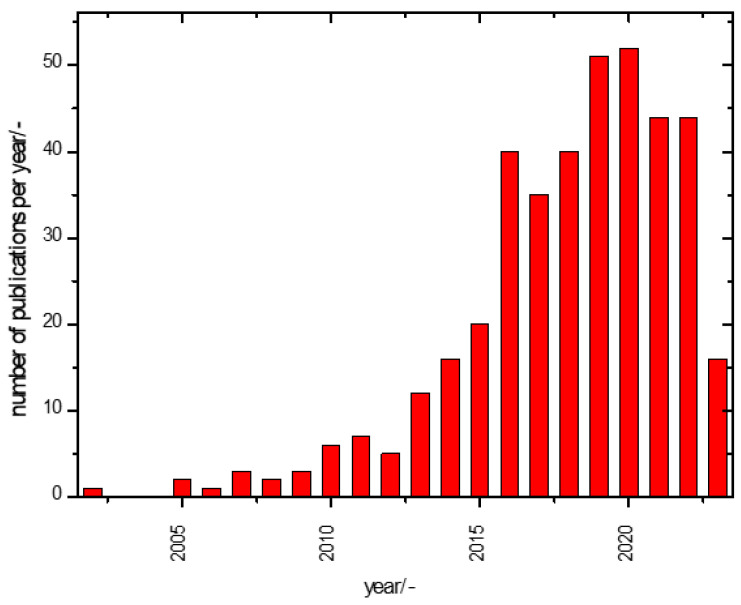
Annual publication numbers of reports with “ageing” or “ageing” or “degradation” and “supercapacitor” anywhere in the title, keywords or abstract (data from Web of Science^®^, retrieved on 30 April 2023). Further publications addressing this topic (mentioning the keyword(s)) somewhere in the text could not be counted; when noticed and considered relevant in the present context, they were evaluated below. The very few publications on “electrochemical capacitors” or “double layer capacitors” instead of “supercapacitors” were included; the associated confusion suggests further systematic use of technical terms. More on this topic can be found in [46,47].

Considering these possible pitfalls, a look at the publication history on the subject of this report is fraught with uncertainties. Figure 1 illustrates, nevertheless, that awareness of the imperfections of supercapacitors, i.e., an imperfect ageing behaviour over time, has existed for years already.

## 2. Causes and Mechanisms

In this section, chemical, electrochemical and related processes observed with electrodes and electrode materials resulting in poorer performance (in whatever terms) are presented and discussed. Because they differ significantly for materials employed in EDLCs and in capacitors employing redox-active materials and their respective electrodes, the text is organized accordingly.

### 2.1. Causes and Mechanisms on the Material and Component Level

Materials deterioration (or degradation) can either be studied with the actual materials of interest themselves or incorporated into an electrode or as part of an actual device. In the latter, specific interactions with an electrolyte solution will become visible; such studies are considered below (see Section 2.2.1).

#### 2.1.1. EDLC-Type Materials and Electrodes

Chemically and electrochemically aged carbon, i.e., exposed to acetonitrile-based electrolyte solutions, without and with electrode potential control at various temperatures, has been studied [48]. Evidence of both chemical and electrochemical reactions at “non inert” carbon surfaces (different types of carbon were studied) and the covalent attachment of most elements found in the electrolyte solution on the carbon surface (more on the positive electrode), yielding the formation of surface functional groups, was noticed. In a subsequent study, the authors identified further degradation contributions, including structural modification of the porous structure, clogging of pore openings and decomposition of cell constituents [49]. Infrared spectroscopy confirmed that performance losses were mostly due to positive electrode degradation [50]. Electrodes with *N*-doped graphene showed performance degradation attributed to surface oxidation as evidenced with X-ray photoelectron spectroscopy (XPS), causing increased Ohmic resistance [51]. The concluded inhibition of electron transfer remains unclear; presumably, electron transport (conduction) in the absence of an electron transfer Faradaic reaction was the intended meaning. The detrimental effects of surface functional groups have been highlighted based on a detailed study [52]. Ageing of an AC derived from carbonized polypyrrole PPy (what is meant by “activation” of PPy remains unclear) has been studied using a combination of electrode potential/cell voltage float and the cycling protocol of both single electrodes and complete cells with an aqueous KNO_3_ electrolyte solution [53]. Essentially, self-discharge and capacitance retention were studied, but neither degradation nor ageing were actually addressed. Activated carbon spheres used in the positive and negative electrodes of EDLC devices showed decreasing mechanical strength with growing current density [54]. Structural degradation as a further effect was tentatively assumed but not demonstrated. Degradation of freestanding electrodes prepared from monolithic carbon spherogels results in loss of the central cavity and sphere degradation of thin-walled samples; therefore, thicker walls are preferable [55]. The particularly detrimental effect of even small concentrations of chloride ions on carbon stability has been highlighted [56]. Ageing and degradation of eight different carbon materials used in the negative electrode of EDLC devices with electrolyte solutions of propylene carbonate or acetonitrile and Et_4_NBF_4_ have been studied [57]. Different from degradation in the range of higher electrode potentials (i.e., of positive electrodes), degradation initially happens on the basal plane, not at edge sites. Degradation of *N*-doped graphene nanoflakes in contact with an ionic liquid electrolyte has been attributed to the intercalation of tetramethylammonium ions between graphene layers resulting in exfoliation [58].

Substitution of fluorine-containing binders by materials from renewable sources is of growing interest. Unfortunately, stability in terms of capacitance retention for electrodes prepared with such binders varies widely, with results not yet being practically viable [59]. Collapse (i.e., restacking or agglomeration in more general terms) of graphene- and MXene-based materials [60] result in capacitance and performance degradation [61,62]; in addition, oxidation may have a negative effect on performance [63]. A similar effect resulting in poorer performance is restacking of carbon nanotubes (CNTs) during fabrication, subsequently hindering ion movement [64]. This type of degradation does not appear to happen during the operation time of a device and is not a form of ageing.

In a study limited to electrodes made from multiwalled carbon nanotubes (MWCNTs) supported on stainless steel, evidence of ageing and degradation similar to that obtained with other carbon materials in contact with aqueous as well as non-aqueous electrolyte solution was obtained [65].

Corrosion of stainless steel current collectors in EDLC-type supercapacitors in particular at the positive electrode has been studied [66], and corrosion protection by a siloxane coating has been proposed.

#### 2.1.2. Battery-Type Materials and Electrodes

As discussed elsewhere [67,68], battery-type electrodes are mostly composed of an active material, a binder and added electronically conducting carbon to make up for the insufficient conductivity of the active material. As single active materials, metal chalcogenides of simple- (e.g., MnO_2_) or complex-composition MeMe1_x_Me2_y_O_z_ (e.g., CoFe_2_O_4_), intrinsically conducting polymers (ICPs), and a few further inorganic materials showing electrochemical redox activity (e.g., transition metal oxynitrides) have been examined. Both organic and inorganic materials have been reviewed extensively. Because of their inherent flaws (especially the insufficient electronic conductivity and stability of many chalcogenides and lack of stability of many ICPs), composites have been prepared and studied and reviews are available [67]. The causes and mechanisms of material degradation of single materials and electrodes, as well as of composites and their electrodes, differ substantially because of the fundamentally different chemistries and properties of the materials. In the case of MnO_2_-based electrodes, the dissolution of manganese ions is a frequently encountered mode of degradation and electrode ageing [69,70,71]. As pointed out, in addition to the formation of actually solvated manganese ions, detachment of particles becoming possibly inactive, redeposition in structurally different forms and further phenomena may occur. Detachment of active material particles (somewhat misleadingly called delamination in [72]) may also occur with composite materials. MnO_2_ combined with cotton fabric into a positive electrode showed some particle coarsening during redox cycling [73]. In the case of MoO_x_ as an active mass, certain redox states of the molybdenum may be prone to instability; thus, the selection of the suitable operating electrode potential window is highly important [74]. Volume change during redox cycling has also been identified as the reason for the electrode degradation of manganese molybdate (MnMoO_4_·nH_2_O); using nanosheets of this material increased stability [75]. An unspecified “phase separation” in composites of carbonaceous materials has been claimed to be a cause of electrode performance degradation [76]. α-Co(OH)_2_ has been claimed to have a higher storage capability than *β*-Co(OH)_2_ but is converted into the latter phase upon redox cycling, resulting in capacitance losses [77]. Formation of *α*-Co(OH)_2_ via hydrolysis of ZIF-67 results in a stable version [77]. The ageing of TiO_2_ nanotubes in aqueous electrolyte solutions has been studied [78]. The results suggest a small dependency of performance degradation on synthesis procedure. Similar observations regarding the effects of synthesis details have been reported for a composite of such nanotubes coated with MnO_2_ [79]. A very specific type of ageing was encountered with catechol-based polymeric redox materials [80]. A few days after reparation, a change of colour of the material already indicated an autoxidation of the catechol groups and subsequent crosslinking associated with a higher redox potential. This “ageing” increased the operating cell voltage and thus its energy density. Studies of ageing and degradation of a composite electrode of reduced graphene oxide rGO/Fe_3_O_4_ operation in a sodium sulphite electrolyte solution revealed the formation of FeS, which showed poor stability during further charge/discharge [81]. Performance degradation of an electrode of Ni_0.34_Co_0.66_Se_2_ nanorods was attributed to selenium losses [82]. Degradation of vanadium nitride, suggested as an electrode material for microsupercapacitors, has been attributed to the formation of oxides on the material surface [83].

The degradation of ICPs, particularly noticeable during electrode potential cycling in, e.g., CVs, has been observed frequently; for overviews, see, e.g., [84,85]. In-depth studies, beginning with the more or less pseudocapacitive behaviour of ICP-electrode-specific degradation processes have been identified. Using Raman spectroscopy, structural changes of PPy on the molecular level, and “the low mechanical stability of the C=C bonds in PPy”, were related to the observed capacitance degradation based on observed band shifts [86]. The decreasing intensity of Raman bands attributed to the bipolaronic state of the oxidized PPy may also indicate a decreasing charge storage capability because of decreasing available redox-active sites caused by the irreversible transformation associated with molecular chain deformation. During charge/discharge cycling, changes of PPy in a composite with rGO were observed with various analytical tools without yielding a coherent conclusion [87]. Electrochemical degradation of a PPy film with *p*-toluene sulfonic counteranions has been studied with various methods [88]. The impedance data suggest the growing Ohmic resistance of the ICP, limiting current flow and thus decreasing effective capacitance; further experimental evidence, such as growth of a carbonyl peak in infrared spectroscopy, is mentioned, but is hard to correlate with the somewhat diffuse experimental procedure with a mix of ex situ storage and in situ electrochemical measurements. A similar conclusion regarding the growing Ohmic resistance of the ICP has been reported elsewhere [89]. In a study of PPy nanowires, dissolution of PPy was observed [90]. Morphological changes of PPy nanotubes evidenced with impedance measurements during galvanostatic cycling negatively affected capacitance [91]. Mechanical degradation of PPy in a PPy/bacterial cellulose composite, as evidenced with SEM, was identified as the cause of the noticed capacitance decrease [92].

The Influence of electropolymerization condition, i.e., pH of the solution, pH-value and presence/absence of dissolved dioxygen on PPy film stability has been studied [93]. Films grown at lower pH-values and in the absence of dioxygen were more stable according to the results of electrochemical impedance measurements. It is possible that dioxygen oxidized the PPy film. Unspecified “degradation” of PPy, evidenced with redox electrode potential shifts and changing currents in CVs, has been claimed [94]. XPS was employed to find the effects of preparation conditions, but apparently not of degradation. Ion trapping in polymer films as a possible contribution towards degradation has been studied in detail [95].

The electrochemical capacity fading of a PANI-based supercapacitor electrode was studied with XPS [96]. Hydrolytic degradation supported by water molecules transported into the polymer during cycling was concluded from the disappearance of the chlorine signal initially caused by the chloride counteranions in the ICP and an increase of the sulphur (from the sulphuric acid electrolyte) and oxygen. This suggests the need for a closer inspection of the water-transport properties of the studied anions beyond the properties already examined [97,98,99]. Based on further evidence from Raman spectroscopy, the degradation products *p*-aminophenol, *p*-benzoquinone and quinone imine created by breaking bonds between repeat units, and further chemical as well as electrochemical reactions, were identified [100]. Degradation of PANI performance has been related to molecular weight decrease, changed state of aggregation and changed morphology [101]. Lower molecular weight is caused by the disintegration of the ICP through bond breaking. The kinetics of PANI degradation aiming to identify optimum operation parameters have been reported [102]. Degradation products of PANI in a composite with cobalt–aluminium-layered double hydroxide were identified as being redox-active, contributing to storage capacity [103]. The identities of these degradation products were not revealed.

PANI shows two clearly separated redox transitions from the leucoemeraldine to the emeraldine and from the emeraldine to the pernigraniline state. Although both transitions are fairly reversible in terms of maintained redox activity, and thus charge storage capability [84,85], PANI in the pernigraniline state is rather susceptible to degradation by, e.g., nucleophilic attack of aqueous electrolyte solution constituents [104,105,106]. This is even more pronounced during overoxidation, i.e., even higher electrode potentials [107]. Accordingly, proper cell voltage control, limiting the positive electrode redox process to the first transition, will help to avoid degradation and thus electrode and cell ageing [108]. The effects of defects on the molecular level in PEDOT:PSS and their influence on electrode ageing have been examined, and a detrimental effect of elevated temperature was noticed [109]. Ageing of a composite material of graphene oxide and PEDOT:PSS formed with glucose as a green filler by ultraviolet radiation has been studied [110]. The effects of the irradiation were not revealed.

The degradation of multilayer electrodes composed of different ICPs has been examined [111]. Multilayers of PEDOT and poly(*N*-methylpyrrole) (PNMPy) were more stable than multilayers of just one ICP. The former kept their porosity even after extensive cycling. Favourable interactions not further specified between the ICP layers were invoked as the reasons for the improvements [112,113]. Layer-by-layer films of poly(*o*-methoxyaniline) and poly(3-thiophene acetic acid) have been examined as supercapacitor electrode material [114]. Films casted only with the formed material showed lower stability and faster degradation, attributed to counterion ingress/egress (see also [115]). The frequently deplored degradation of PANI was mostly attributed to swelling/shrinking during cycling [67], as well as to structural degradation on a molecular level [116], which has also been observed with composites employing this ICP, e.g., in particular at elevated temperatures with PANI/MnFe_2_O_4_ [117]. Extra-large capacitance values of PANI/graphene composites have been attributed to the redox activity of oligoaniline PANI degradation products [118].

In composites, slower degradation and consequently slower ageing have been attributed to inhibited dissolution of, e.g., MnO_2_ when coated with PANI [119]. The inherent flaw of this ICP, its degradation caused by swelling/shrinking during cycling, is ameliorated by keeping the coating of PANI on the MnO_2_ nanowires very thin. A long-term stability claim for a composite of PANI and MoS_x_ after 150 cycles (!) appears to be slightly overoptimistic [120].

Performance losses of metal chalcogenides and of the electrodes incorporating them can be attributed to various modes of deterioration. Structural changes during cycling, i.e., during changes of the redox states of the metal ions in a chalcogenide and the associated insert/egress of further cations, can be at least partially irreversible. In the case of hollandite α-MnO_2_ DFT studies, the insertion of alkali metal cations causing distortions of the unit cell and changes of the manganese coordination are identified as possible causes of degradation [121]. Performance loss, and in particular capacitance loss, of Ni(OH)_2_-based electrodes appears to be caused by dissolution of the nickel hydroxide, and consequently measures to inhibit dissolution by, e.g., coating with an ICP may be a remedy [122]. Such unwelcome structural changes and thus degradation have also been observed with iron oxides [123].

Negative electrodes based on Li_4_Ti_5_O_12_ suggested for lithium-ion capacitors (LiC) showed degradation via lithium ion trapping at the octahedral 16c position of the titanate [124].

### 2.2. Causes and Mechanisms at the Device Level

The same arguments provided above when describing the reasons behind the organization of materials will be applied again in the following section at the device level. Because processes may be considerably affected by the chemical identity of the solvent in the electrolyte solution, the separate handling of devices with organic (solvent) electrolyte solutions which is occasionally observed is reflected below by a separate section focusing primarily on this aspect.

#### 2.2.1. Devices with EDLC-Type Electrodes

Temperature, particularly elevated temperature, and too-high cell voltage have been identified repeatedly as the main sources or at least major contributors of ageing [125,126,127,128]. High-voltage supercapacitors with aqueous electrolyte solutions operating at cell voltages > 1.23 V may show gas evolution due to carbon electrode decomposition (mostly oxidation at the positive electrode) and hydrogen evolution at the negative electrode [129]. Using cell pressure measurements and online electrochemical mass spectrometry, it was confirmed that oxidation may begin at cell voltages as low as 0.6 V, whereas noticeable hydrogen evolution via water decomposition starts around 1.6 V. During short-term cycling, some reversible gas formation/consumption was observed. During long-term cycling, irreversible side-reactions begin which are associated with increased cell pressure and performance deterioration. Based on the reported observations, cycling was found to be more harmful for electrode integrity than keeping a fixed cell voltage; this apparent contradiction to the opposite conclusions presented above may be related to the fact that in the study discussed above [7,8], only capacitance retention was monitored, not electrode integrity or cell pressure. In a similar study with an aqueous Li_2_SO_4_ electrolyte solution, similar observations were made [130]. The positive electrode caused most of the ageing of the device; it had many more surface-oxygenated functional groups than the negative electrode. These groups contributed to pore blocking and associated loss of electrochemically active surface area. With this solution, an upper safe cell voltage of 1.5 V was concluded, significantly higher than with KOH or H_2_SO_4_. In a similar study with a LiNO_3_-based aqueous electrolyte solution, essentially the same results regarding degradation and cell ageing were obtained [131]. Experimental options of mass spectrometry in similar studies have been described [132], and a supercapacitor cell for operando GC-MS has been developed [133]. As an alternative, in situ Raman spectroscopy of gas evolving during cell operation has been applied successfully [134].

Minor differences in ageing, in particular under accelerating conditions between electrolyte solution using different solvents (e.g., acetonitrile and propylene carbonate), have been observed [126]. The influence of the carbon material became very obvious in a comparative study with EDLC devices and an organic solvent-based electrolyte solution with two different carbons [135]. One carbon showed continuous degradation of both ESR and capacitance, whereas the other one initially showed only a growth of ESR. The latter behaviour was explained by invoking the formation of a passivation layer. Unfortunately, the characterization of the two carbons did not include an examination of the carbon surface chemistry; thus, it can only be assumed that such differences may cause the different ageing behaviour. Structural details may also be relevant; the first carbon had a larger specific surface area, presumably due to a larger fraction of micropores more susceptible to clogging as observed with the capacitance decrease. In a subsequent study, again comparing two electrodes, which, in addition to data in the earlier study were characterized in terms of different numbers of surface functional groups, further details regarding an ageing mechanism were proposed [136]. The larger number of surface functional groups contributed to passivation layer formation. With both carbons, the presence of traces of water in the cell contributing to the proposed formation of a superacid HF·BF_3_ as the first step at the positive electrode was stressed. In a review of analytical techniques for supercapacitor material characterization, the detrimental effects of surface functional groups on carbon materials for EDLC devices was stressed [137]. Appropriate methods for evaluating the efficiency and capacitive behaviour of supercapacitors have been critically examined; the different types of devices addressed in the title are presumably just different brands of EDLC devices [138]. References to redox reactions, the addition of KI and di-hydrogen evolution may indicate that, beyond EDLC devices, systems with Faradaic charge storage were also included. The need to distinguish between Coulomb and energy efficiency is stressed, and reporting of the latter is highly recommended. Sensitivity of devices with aqueous electrolyte solutions to cell voltage applied during cycling was confirmed [139], but only floating at 1.5 V yielded the highest capacitance; at 1.6 V and even moreso at 1.8 V, significant degradation was observed.

Capacitance losses during operation are referred to as degradation, particularly in earlier reports. As a consequence, capacitance losses during operation (or testing) which can be reversed by keeping the device at zero voltage for some time have been referred to as reversible degradation [140]. Processes and mechanisms enabling this were not reported; a possible connection to incomplete discharge was indicated. 

A microsupercapacitor with 10.8 V operating voltage has been described [141], and was constructed as a series connection of nine cells of the EDLC type with graphene electrodes prepared by laser writing on a polymer film support. After 100,000 cycles, 100% capacity retention was observed, but reasons for the stability were not provided. In a rare exception, the strength and ductility of completely reduced GO and the good interfacial adhesion between it and the also employed MnO_2_ are suggested [142].

#### 2.2.2. Devices with Battery-Type Electrodes

Supercapacitors with ICPs as active masses can be prepared in various configurations, as previously discussed in detail [67,68]. Although a symmetrical configuration with, e.g., PANI both as a positive and negative electrode is hardly preferable, it has been tested with respect to electrode degradation (and implicitly cell ageing) [143]. Changes in terms of charge storage capability, Ohmic resistance and charge transfer resistance of any electrode reaction at the positive electrode were much more pronounced than at the negative electrode. Consequently, an asymmetric device with PANI as the negative and AC as the positive electrode were built and successfully tested for good capacity retention during cycling. Charge trapping in the negative electrode of a symmetric device with *p*/*n*-dopable conducting redox polymers has been found to be the main reason for performance degradation [144]. Partial recovery of charge trapping by potential cycling was found to be possible.

#### 2.2.3. Hybrid Devices

LiCs combining a negative lithium intercalation electrode and a positive EDLC electrode have been subjected to post-mortem analysis after accelerated ageing tests at ambient and strongly elevated temperatures [145]. Pore blocking of the positive AC electrode and lithium-ion loss of the pre-lithiated negative electrode were found to be the major factors contributing to device ageing. Detrimental effects of the AC (positive electrode) surface functional groups possibly adsorbing lithium ions were indicated as an important subject of further research. Because of the ageing of LiCs, the effects of cell voltage are different from those found in EDLC-type devices [146]. The particularly fast ageing at low cell voltages was considered in an adapted model based on the results of electrochemical impedance measurements. Calendar ageing of LiCs at elevated temperatures both fully charged and discharged has been studied using mostly electrochemical impedance measurements [147]. Fully discharged cells suffered huge capacitance loss compared to the charged cells. The low operating potential of a graphite electrode in an LiC has been claimed to be a cause of lithium plating on the graphite surface and consequently performance degradation [148]. With granular Li_4_Ti_5_O_12_ as the negative electrode in an LiC, performance degradation due to gas evolution (H_2_O and HF) was found to be proportional to applied current density [149]. Further inspiration may be gained from observing high-power lithium-ion batteries with a performance claimed to be equivalent to many applications currently assigned to supercapacitors [150].

#### 2.2.4. Devices with Organic (Solvent) Electrolyte Solutions

Causes of EDLC-type supercapacitors ageing with organic electrolytes (this abridged but over-simplified description is popular, and is used here for conciseness) and AC electrodes under voltage floating conditions (2.5 V at 4000 to 7000 h) have been studied previously [151]. Using several common analytical methods, various decomposition products were identified. Differences in the amount and identity of positive and negative electrodes suggested redox reactions between the electrolyte and surface functionalities on the carbon. In addition, these decomposition products plugged some pores of the carbons, as evidenced by BET measurements. Diminished accessible surface area and poorly conducting deposits were invoked as explanations for the increased ESR and decreased capacitance of the studied devices. Lower concentration of surface functionalities was suggested to be favourable for slower ageing. Gas pressure increase of cells under accelerated ageing conditions was compared for cells with acetonitrile-based electrolyte solutions containing different ammonium tetrafluoroborate salts [152]. Cells containing an electrolyte with an acyclic cation showed a much larger pressure increase, attributed to weaker solvent–electrolyte interactions. The influence of different organic solvents on gas pressure evolution has been examined [153]. With *γ*-butyrolactone, gas evolution started at 2.5 V cell voltage, whereas gas evolution was small even at 3.25 V. During and after ageing at elevated temperatures, gas pressure changes and the elemental composition of electrodes and their changes for an EDLC device with an electrolyte solution of acetonitrile and triethylmethylammonium tetrafluoroborate were examined [154]. An additional “precharge at low voltage” resulted in smaller pressure rise at high voltages, and a large pressure increase was observed at 3 V cell voltage, well above the rated operating voltage. On the electrode, deposits were found which explained the increase of ESR and decrease of capacitance.

Ageing and failure modes of EDLC devices under constant load (different from the well-established meaning of this term in battery testing, where it suggests a constant Ohmic discharge resistor connected to the battery, the complex current–time–charge–discharge program applied here is very much different) have been studied [155]. Capacitance, ESR and leakage current were examined. At elevated temperatures (>70 °C) and high cell voltage, failure of devices caused by internal pressure build-up were observed. Ageing at elevated voltages (3.3 V) resulted in changes in the recorded impedance data, suggesting an increase of electrode surface in heterogeneity not observed during ageing at elevated temperatures. Leakage current decreased during constant voltage test, and therefore cannot be taken as an indicator of device ageing. Capacitance losses below the commonly accepted 80% value as an end-of-life criterion always occurred earlier than the doubling of the ESR assumed as the other criterion. EDLC supercapacitors with an acetonitrile-base electrolyte solution of alkylammonium fluoroborate studied by excessive overcharge released several decomposition products of all constituents [156]. The formed HF attacked the aluminium foil used as electrode support. Tests of similar systems under less abusive conditions revealed the destruction of the adhesive layer between the current collector and active material to be a cause of performance degradation and device ageing [157]. In further accelerated life testing of such devices, the safety and reliability of EDLC devices were tested, along with excess voltage and elevated temperature conditions [158]. Decomposition of acetonitrile from the electrolyte solution and destruction of the electrodes caused by the continuous stress were observed. An attempt to identify indicators of over-voltage and over-temperature stress has been reported [125]. As fault indicators, the equivalent series resistance, capacitance and cell voltage relaxation after disconnection from power sources were identified [125].

Physical damage observed in post-mortem analysis of cells and their components were more pronounced with voltage-stressed than with temperature-stressed cells. Further studies of such devices revealed damage to the positive electrode at cell voltages above 3.0 V caused all of the observed performance degradation, and at voltages above 3.5 V the negative electrode also contributed somewhat [8]. More degradation products were found on the positive electrode, porosity of the electrodes decreased. The influence of the applied currents and voltages on the ageing of a commercial supercapacitor (NESSCAP 10 F) with acetonitrile-based electrolyte solution has been studied [159]. At currents > 4 A over 60,000 cycles, damage to electrodes was noticed. With an excess voltage > 3.2 V, the device was destroyed. At high voltage, the binder was destroyed and the current collector was covered with a passivating film, causing increased contact resistance according to post-mortem analysis of the electrodes. At higher currents, only minor damages of this type were found. Morphological changes were also more pronounced with higher voltages.

An accelerated ageing test of EDLC devices with an ionic as the electrolyte combining cycling and “floating at high potential” (obviously “voltage hold”) yielded > 80% capacity retention after 100 h (!) [160].

The ageing of a supercapacitor with a redox-active component (KI) added to the electrolyte solutions was examined [161]. GCD had a more degrading effect than voltage floating tests on carbon structure, but voltage floating tests were more detrimental overall. In a redox flow capacitor with a membrane separator, membrane fouling, also seen in flow battery studies [162,163], was identified as the reason for power degradation and device ageing [164].

Deep eutectic solvents (DES) have been proposed for supercapacitors operating at elevated temperatures [1]. Because one component (in [1], it was acetamide) may evaporate, precipitation of the other component (LiNO_3_) may occur, yielding faster ageing because of the detrimental effects of solid deposition on porous electrode performance as discussed above. A DES based on lithium bis(fluorosulfonyl)imide and formamide as the electrolyte in an EDC-device showed gas evolution at excessive cell voltages, which was in part electrochemically reversible [165].

To enable lower operating temperatures, EDLC cells with a mixture of water and methanol as the electrolyte solution solvent have been proposed [166]. The observed ageing was attributed to oxidation at the positive electrode and corrosion of the stainless steel current collector. Given the frequently stressed sensitivity of EDLC devices vs. elevated temperatures, attempts to select cell constituents suitably were reported after identifying ageing and the reasons for modified device failure at 120 °C: fusion of the separator increasing the ESR, decomposition of the separator and delamination of active masses from current collectors [167].

#### 2.2.5. Devices with Solid Electrolytes

Given the numerous advantages of solid or at least semi-solid (gelled electrolytes), further degradation processes inside the ionically conducting phase between the electrodes may contribute to device aging. In the case of an anionically conducting polymer, a decrease of ionic conductivity somehow associated with some not yet resolved change in the polymer was identified as a cause of capacity decrease [168].

The accelerating effect of elevated temperatures on performance degradation was also confirmed for a CNT-based all-solid-state supercapacitor [169].

A symmetric device with two PPy electrodes and an ion liquid-based gel polymer electrolyte showed substantial ageing in terms of capacitance loss, attributed to the conceivable formation of a passivation layer between the electrode and electrolyte [170].

#### 2.2.6. Flexible, Stretchable and Bendable Devices

In addition to supercapacitors employed in electric and electronic circuits of traditional design and construction, new applications for flexible, wearable and bendable devices sometimes demonstrate further functions, such as transparency or electrochromism. For such devices, further causes of performance degradation must be considered.

In a stretchable electrode, the detrimental effects of stress induced by stretching could be reduced by using a composite of PPy and MnO_2_ nanosheets as the active material instead of MnO_2_ alone [171]. With PPy alone as the active material, stretching-induced degradation was also low; this was attributed to the various structural advantages of the polymer. Unfortunately, PPy, as with many other intrinsically conducting polymers, has other flaws, such as active mass in supercapacitors. For overviews, see [67,68].

Mechanical degradation by bending, folding, flexing or other forms of mechanical deformation may cause degradation of device performance identified as capacitance loss. In reports, high stability, i.e., minor degradation of a given material and device are frequently stated, but the reasons for this are not provided.

## 3. Modelling

For many users, the reasons for performance losses on the molecular or electrode level are of minor interest only because the user’s interest is for obvious and quite natural reasons, focused on device performance and change of this performance (ageing) during use. For practical reasons, particularly for the prediction of device performance development during further use, modelling is frequently applied. Although an understanding of device operation and behaviour on all levels may be helpful, it might not be requested from a user interested in predicting future device performance [172]. Thus, many models, in particular those developed within electric and electronic engineering, are purely descriptive–empirical. Because they can possibly be implemented into auxiliary electronics in devices containing a supercapacitor requiring monitoring, such models may be actually helpful in the safe and successful application of a supercapacitor even without understanding why the supercapacitor degrades and finally fails. Formulation of predictive laws may be even more empirical when taking into account experimental observations and general chemical knowledge, such as those of chemical kinetics or other physical evidence. An ageing law links ageing kinetics with the growth of an interfacial layer (somewhat confusingly called solid electrolyte interface (SEI), a term applied so far only in reactive metal batteries) covering more of the electrode surface with time [173]. Using a broad array of samples, experimental data could be fitted to predictions of the proposed ageing law; further development aims to realize the inclusion of temperature and cell voltage. A further model for the prediction of supercapacitor ageing in vehicular applications has been reported [174]. Based on electrochemical impedance measurements during accelerated ageing tests of EDLC supercapacitors, an ageing model has been developed [175,176]. An improved multipore impedance model has been developed to aid in the interpretation of floating ageing measurements [177]. An overview of supercapacitor modelling is available [4], while models with particular relevance to self-discharge have been discussed elsewhere [178].

As most proposed supercapacitor models are electric models valid only early in their lifetime, a more complex model taking into account changes during lifetime has been proposed [11]. Modelling as a support for lifetime behaviour prediction for EDLC supercapacitors has been reported based on cycling tests under various experimental conditions [179,180]. Linear capacitance retention trends could be extrapolated linearly with temperature as the main factor. A life-cycle prediction model combining extrapolation and acceleration factors was established. The effects of voltage and temperature on leakage currents of EDLC devices measured during calendar ageing have been studied and used in a leakage current model [181]; further considerations of leakage current and self-discharge have been reviewed elsewhere [178].

Supercapacitor modelling has been reviewed encompassing further engineering aspects [182]. State-of-health estimation based on such modelling has been attempted, starting with the results of degradation/ageing studies taking into account temperature, voltage and cycling conditions. Given the major influence of temperature and the considerable impact of current in actual operations, models taking these contributors into account have received particular interest. Based on a review of microscopic changes attributed to these driving forces and their influence on macroscopic performance indicators [183], a model for predicting the state of health, in particular the kept capacitance, was developed and validated for a time period of 450 h by comparing measured and predicted capacitance values [184]. Using an electric model and experimental results of calendar ageing studies, a method for supercapacitor online health diagnosis has been developed [185].

Balancing based on multivariable modelling of multi-cell setups can help mitigate the accelerated ageing caused by temperature differences between cells [186] (in such arrangements, a 10 °C difference can easily be observed between cells without precautions; see also [176]). A model particularly taking into account the ageing effects of supercapacitor cycling in vehicular applications and at elevated temperatures has been reported [187]. An algorithm method for predicting the remaining lifetime of supercapacitors has been developed [188]. In vehicular applications, fuel cells are frequently combined with supercapacitors with an energy management strategy that controls the contributions of the various components [189]. The ageing effects of the components may affect utilization of the hydrogen fuel consumption negatively; accordingly, ageing models are incorporated into the strategy. In addition to combining a supercapacitor with a fuel cell, such combination with secondary batteries, in particular lithium-ion batteries, may help to improve the overall performance of the complete system and to slow down ageing [190,191].

Parametric model structures possibly suitable for EDLC diagnosis have been compared [192]. In lithium-ion battery degradation studies, the supervised learning of synthetic big data has been proposed [193]. This approach may be applicable in supercapacitor ageing studies as well.

A reduced-order physics-based model has been developed to support systems combining batteries and EDLC-type supercapacitors [194]. For comparison and further inspiration, ageing models developed for lithium-ion batteries have been considered [195,196,197,198,199]. Descriptions and models of performance and ageing of electrochemical energy storage and conversion devices, including EDLC supercapacitors based on thermodynamics, have been developed [200].

An overview on modelling of supercapacitor ageing [201] is available.

## 4. Countermeasures

### 4.1. EDLC-Type Supercapacitors

The ageing of supercapacitors is significantly accelerated at elevated temperatures. Consequently, temperature management with a heat-transfer and -removal (cooling) capability to address even larger heat evolution in the later stages of the lifetime of a device has been strongly recommended [11]. The use of phase-change materials as a possible contribution to heat management has been proposed [202].

EDLC-type supercapacitors are subject to degradation processes even in an only partly charged state, particularly at the positive electrode. Because surface functionalities (in particular oxygen-containing ones) on the carbon electrode participate by redox reactions with the organic electrolyte solution, carbons with small surface concentrations of such functionalities appear to be advantageous. The loss of redox storage capability because of the missing redox capabilities of such functions may be acceptable; however, it appears to be a rather unreliable and unpredictable contribution. The further detrimental effects of such surface functional groups, such as lithium ion adsorption, further recommend the careful control of their presence in carbon materials for supercapacitor electrodes. Careful and controlled application of nitrogen-containing functional groups has been found to enhance performance and slow down structural electrode degradation [203]. *N*-doping of carbon materials may also improve the stability of such material in an ionic liquid electrolyte when operated at elevated potential/voltage [204]. In addition to a cautious consideration of surface chemistry, porous structure and pore size distribution should be considered. Moderate fractions of micropores and consequently somewhat lower specific surface area may be beneficial for slower ageing based on observations in a comparative carbon study discussed above [135]. From the observation that electrode degradation proceeds even at voltages much smaller than the rated values for a supercapacitor, it has been recommended to keep devices in a partly charged state or even to discharge (reset) them fully from time to time. This procedure creates just the opposite effect and cannot be recommended [9]. Coating of the electrode material via atomic layer deposition (ALD) of a thin layer of Al_2_O_3_ just a few nanometres thick resulted in protection of the surface functional groups and slowed down electrolyte solution degradation [205]. Similar effects were achieved with an alucone coating on LiCoO_2_ intended for lithium-ion batteries [206]. With NaPF_6_-based electrolyte solutions during thermal degradation, PF_5_ may be formed, which can cause autocatalytic degradation of the solution [207]. The addition of hexamethylphosphoramide will bind PF_5_ and thus slow down degradation.

In cases of aqueous electrolyte solutions, carbon corrosion via carbon oxidation of the positive electrode in the presence of water may be substantial once the potential of the positive electrode approaches the potential of water oxidation. Careful cell voltage limitation by any means, ranging from protection diodes inserted in parallel to the cell to more complex supervision circuits, will help to avoid unwelcome high cell voltages. Given the repeatedly stated dominance of positive electrode degradation, suitable mass balancing may provide an option to extend the useful lifetime of a supercapacitor with the use of more mass in the positive electrode [50]. This confirms earlier observations and suggestions [208]; unfortunately, this contribution was later ignored [50]. Later attempts towards cell optimization using determination of the stable cell voltage window, taking into consideration appropriate electrode mass balancing, confirmed this concept once more [209]. Using additives in the electrolyte solution to slow down ageing has been studied [210], starting with the well-established oxidative attack at the positive carbon electrode tocopherol (vitamin E) with known anti-oxidative properties. In a floating voltage test, a significant extension of the useful lifetime of the device was recorded. The addition of an electrolyte with a redox-active polyfluorinated boron cluster anion [B_12_F_11_H]2^−^ acting as a redox shuttle mitigating solvent degradation during operation under harsh conditions has been proposed [211] (for similar applications in lithium-ion devices, see also [212,213]). A graphene-coated aerogel of CNTs provided a highly compressible and stable electrode material for EDLC devices [214]. The collapse of graphene- and MXene-based electrode materials can be avoided by ion-induced formation of hydrogels [61]. Another option for this purpose is the formation of a composite with MWCNTs [62]. Improved stability in addition to enhanced performance with Mxenes has been achieved with cyclocrosslinking with polyphosphazene [63].

A supercapacitor with a water-in-salt electrolyte and a coating with a composite of Zn and Zn_4_SO_4_(OH)_6_·H_2_O (ATDS) on the negative electrode enabled a potential shift of the positive carbon cloth electrode into a stable electrode potential range [215].

Performance degradation of devices with water-in-salt electrolytes at low temperatures due to precipitation of salts can be inhibited by the addition of acetonitrile, which changes sodium ion solvation [216].

The use of heteropolytungstate acid (H_3_PW_12_O_40_) instead of sulfuric acid as an electrolyte permits the use of stainless steel current collectors instead of more expensive noble metals while keeping high ionic conductivity and capacitive performance [217].

Delamination of the electrode mass from the current collector has been identified as a major degradation phenomenon as discussed above. Laser micro-structuring of aluminium foils has been suggested as an option for improved adherence, better performance and slower degradation [218].

Heat generation inside a supercapacitor via flowing current may be limited by adding temperature-responsive polymers to the electrolyte solution [219]. Once a critical temperature given by the actual properties of the electrolyte solution is reached, the actual capacitance of the device is reduced due to various polymer-related processes, subsequently limiting current flow and further heat generation. Observing a lower cell voltage limit has been found to be beneficial to slow down the ageing of screen-printed supercapacitors with aqueous electrolyte solutions [220]. Various liquid additives to a solvent mixture of acetonitrile and ethyl acetate have been examined for an extended range of operating temperature without stability loss [221].

In arrangements of several supercapacitors connected in parallel for higher current and in series for higher voltage, imbalances between cells may cause excess voltages (i.e., overcharge) of cells, resulting in the damages discussed above (Section 2.2.1. and Section 2.2.3) at too-high cell voltages. Electronic balancing circuits are provided and their effects on degradation has been examined [222]. An active balancing circuit for a two-cell arrangement was found to be beneficial both in terms of better capacity retention and lower ESR increase than without such a circuit.

Recording parameters such as capacitance and ESR related to state-of-health and ageing commonly require the shutdown of the device. Options to obtain these parameters online have been developed [223]. The concept was developed further into an online observer facility [224]. Another option for state-of-health monitoring may be online extraction of cell ESR values as a method of cell and module diagnosis [225].

### 4.2. Supercapacitors with Battery-Type Electrodes

Poor rate performance because of low electronic conductivity and material degradation due to volume changes during redox cycling observed with metal oxides proposed as active masses could be ameliorated by composition with biomass-derived and thus *N,S*-doped AC [226]. More frequently, nanostructuring (nanosheets, nanocones, nanowires, etc., and their arrays or similar arrangements) has been proposed as a remedy [67]. Reducing the particle size of active chalcogenides (e.g., manganese-doped *α*-Ni(OH)_2_ from micrometres to nanometres helped to reduce detrimental inhomogeneity across the particles [227]. A buckled sandwich electrode structure permitted the use of composite material combinations of CNTs and metal chalcogenides with increased stability due to structural mitigation of internal stress caused by ion egress/ingress when redox cycling while accelerating ion transport [228]. Metallic nickel and iron encapsulated in an onion, such as carbon obtained via pyrolysis of the respective phthalocyanines, were effectively protected against contact with the electrolyte solution and did not participate in charge storage (!) [229].

Performance deterioration caused by degradation of layered *δ*-MnO_2_ during cycling could be mitigated slightly by doping with Al3^+^ up to *δ*-Al_0.06_MnO_2_ [230]. The poor electrode kinetics of MXenes caused by sluggish ion diffusion within the too-narrow interlayer spaces and subsequent conductivity degradation can be alleviated by using PPy-coated electrospun polyvinyl alcohol fibres [231]. The beneficial effect of careful voltage/potential control for slower ageing of MnO_2_-based electrodes has been addressed in general terms [232]. When a material is available in several sufficiently redox-active crystalline types (e.g., Ni_x_Fe_3−x_O_4_), a flexible spinel framework seems to be beneficial for higher stability [233]. Seamless graphidyne layers on metal oxides have been proposed as a way of mitigating degrading effects of structural and interfacial instabilities of metal oxides in electrodes [234].

A vulcanization treatment of PANI introducing sulphide or disulphide bonds establishing crosslinks to ameliorate the detrimental effects of volume changes during redox cycling improved stability [235]. At the device level, material architectures taking into account the flaws of specific materials may help to avoid the negative effects of these flaws, thus contributing to increased stability and slower aging. A sponge of CNTs coated first with a thin layer of PPy, then with PANI, ameliorated the drawbacks of swelling/shrinking of the ICPs during cycling, as well as the negative effect of insufficient electronic conductivity of the ICPs [236]. The inverted sequence of ICP coatings provided the highest capacitance retention. Why the ICPs were called pseudo-polymers remains as mysterious as the claim of improved cycling stability absent a comparison material. Another option to alleviate degradation of PANI caused by its brittleness and the negative effects of swelling/shrinking by depositing PANI on polyurethane nanofibers has been suggested [237]. The stability of PANI during cycling was improved by adding some AC (0.04 mass fraction) [238]. A similar observation has been reported earlier without providing compositional details (fraction of AC) and reasons for the improved stability and performance [239]. The function of the added carbon has been discussed elsewhere before [67,68]. In a somewhat similar approach, highly porous carbon obtained from tires as a precursor was used as a scaffold for PANI formation; the improved stability (as compared to plain PANI) was ascribed to the confinement of the ICP in the porous carbon structure [240]. Adding sulfonated carbon to PANI slowed down PANI degradation [241]. Reasons for this have not been provided; it is possible that the insertion of the sulfonated material is similar to self-doped PANI [242,243] in terms of reducing the need for counterion ingress/egress with the associated structural stress. Negative volume change effects and hydrolytic degradation observed with PANI could be avoided by using crystalline tetraaniline flakes [244]. An unspecified “synergistic effect between nanofibers (of poly(vinyl alcohol-co-ethylene)) and PANI” enabled improved stability [245]. With tetraaniline instead of PANI, the higher stability of an electrode with rGO was achieved, which showed self-healing by reestablishing electronic contacts in the active mass after volume-change-induced loss of electronic contact [246].

ICPs with slow hydrolytic degradation, such as polyindole and its copolymers, with, e.g., pyrrole, may be preferable in terms of slower device ageing [247,248]. To avoid hydrolytic degradation of PANI entirely, the use of nonaqueous electrolyte solutions has been proposed [101]. The stability of nanowires of PPy with *p*-toluene sulfonate as the counter anion could be increased by keeping the initial degree of overoxidation small and by keeping dissolved dioxygen out of the device [90]. The negative effects of volume change of an ICP can be avoided completely by using a soluble form of an ICP, e.g., PANI made soluble by using a superacid counter anion (trifluoromethyl sulfonic acid) [249]. The remarkable stability in terms of capacitance retention may be reconsidered when measuring the self-discharge of the device not studied here. Self-discharge of supercapacitors with dissolved redox systems may cause performance limitations as discussed elsewhere [178]. Organic (molecular, oligo- and polymeric) materials, as discussed previously for applications in secondary batteries [250,251] beyond ICPs, may also be of interest for supercapacitors. Ageing behaviour and degradation processes highly depend on structural details of the molecules, and methods of slowing degradation down have been discussed in a typical way in [252], where a decrease of conjugation resulted in improved stability.

In composite materials for flexible/stretchable electrodes, the incorporation of ICPs appears to be a promising approach to slow down material degradation and in effect device ageing [253]. Even without this constituent, a highly flexible and bendable electrode made of carbon cloth decorated with CoS_2_ showed significant stability, presumably because of the very good adhesion of the selenide to the carbon fibre surface [254].

The structural degradation of NiCo_2_S_4_ claimed to be the cause of performance losses of this capacitor electrode material could be ameliorated by encapsulating it into ultrathin graphene shells [255]. Nanocubes of NiCo_2_S_4_ anchored on nitrogen-doped hollow carbon spheres have been suggested as another option for this chalcogenide [256]. Reduced graphene oxide combined with electrochemically prepared Ni(OH)_2_ helped to suppress detrimental microstructural changes during the redox cycling of the electrode [257]. Bio-waste-derived carbon quantum dots composited with nickelpyrophosphate Ni_2_O_2_O_7_ served a similar purpose in improving stability by ameliorating structural stress during charge/discharge [258]. A nitrogen-doped carbon on millerite (nickel sulphide) served a similar purpose, achieving better stability [259]. The hierarchical porous structure of *α*-Ni(OH)_2_ nanosheets was claimed to be a major reason for the remarkable stability of this material in a supercapacitor electrode [260]. Similar observations are reported for Co_3_O_4_; suitable porosity buffering volume changed during redox cycling, resulting in slower degradation and ageing [261]. Whether 10% capacitance loss after 250 cycles with an amorphous Co_3_O_4_ prepared with a structure-directing hexametaphosphate calgon suggests a sufficiently high stability remains questionable [262]. Hollow nanoarchitectures have been proposed as a versatile option for increasing the stability of chalcogenide electrodes [263]. Metal–organic frameworks (MOFs) as precursors of metal chalcogenide may result in compounds with improved stability [264]. When Fe_3_O_4_ is used in an electrode, its degradation via the formation of FeS (see above) can be inhibited by adding FeMoO_4_ to the electrolyte solution [81]. Another option is to coat Fe_3_O_4_ with PANI, which improves both stability and performance [265]. Chemical bonding between PANI and WO_3_ was claimed to be a reason for improved stability [266].

Colour changes of electrochromic electrode materials may be used to easily monitor the state of charge of an electrode and consequently of the device, helping to avoid overcharge and slow down ageing [267]. The detachment of chalcogenides present in particulate form in an electrode may be mitigated by coating with an ICP, such as with PPy on particles of MnO_2_ [72]. Taking into account tip-charge effects with nanostructured materials, this appears to be a rational approach for designing MOF-based electrode materials [268].

### 4.3. Hybrid Supercapacitors and Other Devices

Operating LiCs at excessive voltages may result in premature ageing because of oxidative decomposition of the electrolyte solution [269]. Using a composite of amorphous carbon and nanocubes of MnFe_2_O_4_ enabled an increase of the cell voltage to 4 V without sacrificing stability. Degradation of devices by bending or flexing may be reverted by self-healing processes and materials [270,271]. The addition of compounds to the electrolyte solution suppressing the effects of elevated temperature and electrode oxidation at high cell voltages has been proposed in general terms [272]. Co-doping of MnO_2_ has been found to improve the performance of this material in a magnesium-ion capacitor by increasing both the electronic conductivity and stability [273]. The enhanced stability of a LiC built with graphite recycled from spent LiBs has been attributed to several factors [274].

Chalcogenide electrodes deposited on a flexible polyolefin skeleton were subsequently ionically connected by a zwitterionic gel polymerized inside the skeleton, showing promising stability [275].

The addition of nanocarbon material resulted in improved thermal stability and subsequently slower ageing of a polyacrylamide gel electrolyte [276].

In electrochromic supercapacitor devices, suitable counterelectrode design, and in particular increased surface area, may result in slower ageing and degradation [277].

## 5. Conclusions

In contrast to the frequently claimed extraordinary stability of supercapacitors, suggesting the more or less complete absence of ageing and degradation, these devices are actually subject to significant ageing, occasionally limiting practical applicability. To avoid or slow down ageing, an understanding of relevant processes at the electrode, device and even module/pack level will help to counteract these effects and even to avoid them. The mechanisms of processes depend on the material and the operating charge storage mechanism, and accordingly ageing may proceed at widely varying rates. Generally, the operation of devices at excessive voltages and elevated temperatures in practically all cases accelerated ageing. Countermeasures depend on the same details as the operating mechanisms, and accordingly options to slow down ageing beyond the obvious (low temperature, moderate and efficiently supervised cell voltage and sufficient balancing in multi-cell packs) depend on the specific system.

## Data Availability

Not applicable.
